# Design and Analysis of a Novel Piezoceramic Stack-based Smart Aggregate

**DOI:** 10.3390/s20226438

**Published:** 2020-11-11

**Authors:** Guangtao Lu, Xin Zhu, Tao Wang, Zhiqiang Hao, Bohai Tan

**Affiliations:** 1 Key Laboratory for Metallurgical Equipment and Control of Ministry of Education, Wuhan University of Science and Technology, Wuhan 430081, China; zhuxin951213@sina.com; 2 Hubei Key Laboratory of Mechanical Transmission and Manufacturing Engineering, Wuhan University of Science and Technology, Wuhan 430081, China; wangtao77@wust.edu.cn (T.W.); haozhiqiang@wust.edu.cn (Z.H.); tanbohai43@163.com (B.T.)

**Keywords:** piezoceramic wafer, Smart Aggregate (SA), Piezoceramic Stack-based Smart Aggregate (PiSSA), Lead Zirconate Titanate (PZT), actuators and sensors

## Abstract

A novel piezoceramic stack-based smart aggregate (PiSSA) with piezoceramic wafers in series or parallel connection is developed to increase the efficiency and output performance over the conventional smart aggregate with only one piezoelectric patch. Due to the improvement, PiSSA is suitable for situations where the stress waves easily attenuate. In PiSSA, the piezoelectric wafers are electrically connected in series or parallel, and three types of piezoelectric wafers with different electrode patterns are designed for easy connection. Based on the theory of piezo-elasticity, a simplified one-dimensional model is derived to study the electromechanical, transmitting and sensing performance of PiSSAs with the wafers in series and parallel connection, and the model was verified by experiments. The theoretical results reveal that the first resonance frequency of PiSSAs in series and parallel decreases as the number or thickness of the PZT wafers increases, and the first electromechanical coupling factor increases firstly and then decrease gradually as the number or thickness increases. The results also show that both the first resonance frequency and the first electromechanical coupling factor of PiSSA in series and parallel change no more than 0.87% as the Young’s modulus of the epoxy increases from 0.5 to 1.5 times 3.2 GPa, which is helpful for the fabrication of PiSSAs. In addition, the displacement output of PiSSAs in parallel is about 2.18–22.49 times that in series at 1–50 kHz, while the voltage output of PiSSAs in parallel is much less than that in parallel, which indicates that PiSSA in parallel is much more suitable for working as an actuator to excite stress waves and PiSSA in series is suitable for working as a sensor to detect the waves. All the results demonstrate that the connecting type, number and thickness of the PZT wafers should be carefully selected to increase the efficiency and output of PiSSA actuators and sensors. This study contributes to providing a method to investigate the characteristics and optimize the structural parameters of the proposed PiSSAs.

## 1. Introduction

Nowadays, more and more civil infrastructure is undergoing condition deterioration due to age-related degradation [1], fatigue [2,3,4], impact load [5], corrosion [6,7], environmental conditions, etc. The challenges of deteriorating civil infrastructure call for developing reliable and cost-effective solutions to structural health monitoring (SHM) of civil infrastructure. In SHM of civil infrastructure, notable research based on advanced algorithms, such as damage index-based damage assessment [8,9], image-based damage locating [10] and artificial intelligence algorithms [11], have been reported to provide solutions to real-time monitoring and early warning of civil infrastructure [12,13] by using various sensors, such as strain gauges [14,15], piezoelectric transduces [16,17], optical fiber sensors [18,19], etc. With advantages of low cost, wide bandwidth [20] and quick time response [21,22], apart from in SHM of civil infrastructure, piezoelectric transducers have also been widely used in other fields, including aerospace [23,24,25,26], transportation [27,28] and energy [29,30], and they have displayed good applicability in both passive and active situations [31,32], such as vibration sensing [33], acoustic emission [34], active sensing and electromechanical impedance-based damage detection [35,36,37].

With a tailored protective shell [38], the piezoceramic-based smart aggregate (SA) extends its survival life and it has been widely used in SHM of concrete structures. SAs are usually embedded in concrete structures during the casting process and usually serve as either a transmitter or a sensor to actively transmit stress wave or detect the wave [39,40,41,42]. After the detected signal is processed, the health state of the structure is evaluated, and early warnings are alerted if the structure is in danger. Recently, cracks under seismic and quasi-static load [43,44,45], concrete strength and water percentage in early age [46,47,48], impact and blast load [49,50], debonding or bond slip [51,52], leakage [53] and corrosion of the reinforced bar [54] in various types of concrete structures, including pipes [55], columns [56,57], beams [58,59] and piles [60] of concrete or reinforced concrete (RC), are detected or monitored with the help of SAs.

A typical SA consists of a single piezoelectric wafer, a waterproof and insulated coating, a protective shell and connecting wires. Since SA was first introduced [61,62], the structure of SA is being continuously improved to satisfy the concerns of SHM in concrete structures. In the first generation of SAs, the piezoelectric wafer was protected by a concrete casted block, and this SA only generated a stress wave in a certain direction [63,64]. In the second generation of SA, the concrete protective shell was replaced by two marble pieces to avoid structural destruction and extend the survival time [65,66]. Moreover, to increase the signal to noise ratio of the detected signal, the piezoelectric wafer was surrounded by a copper shield before it was assembled in the marble shell. To excite stress wave in a two-dimensional space omni-directionally, the piezoelectric wafer in the first and second generation SAs was replaced with a piezoelectric tubular cylinder, and this tubular SA has been used to successfully locate damage in two-dimensional concrete slabs [67,68,69]. A spherical SA is developed by Kong et al. [70,71] to generate or receive stress wave omni-directionally in a three-dimensional space. In this spherical SA, the traditional single piezoelectric wafer is substituted by a piezoelectric spherical shell, and both the simulation and experimental results verify that the spherical SA has an excellent actuating and sensing performance. Unlike these aforementioned SAs, a special SA with piezoelectric wafers working in d15 mode is designed to detect shear stress in concrete structures [72]. Moreover, to reduce costs of the traditional SAs due to cable laying, a Zigbee-based wireless SA sensor is designed for SHM in concrete structures; however, the stress wave is still excited by a traditional wired SA transmitter [73]. Moreover, to better understand the electro-mechanical characteristics of SAs, Wang et al. [65] developed a theoretical model to study the performance of the SA with marble protection based on the piezo-elasticity theory [74,75,76,77].

The development of SAs mainly focuses on how to transmit or sense stress waves uniformly in omni-directions or how to avoid functional destruction and prolong the lifetime of SAs. However, how to increase the efficiency and output of SAs is seldom investigated. Recently, the piezoelectric stack is widely used as actuators to generate force or displacement for precision control of, e.g., MEMS, robots [78] and optical instruments [79]. In the stack actuator, many individual piezoelectric wafers are connected mechanically and electrically to increase the displacement or force output. However, the stack is seldom used as sensors to detect signals, and especially the piezoelectric stack has never been previously employed for SA actuators and sensors. Moreover, due to the strong scattering characteristics of concrete structures [80], elastic stress waves are easily attenuated during propagation. Therefore, SAs with large output are helpful for SHM of large concrete structures. In this paper, a novel piezoceramic stack-based smart aggregate (PiSSA) is developed to increase the output of SA actuators and sensors. In the proposed PiSSA, the single piezoelectric wafer in the traditional SA is replaced by a piezoelectric stack, where the piezoelectric wafers are electrically connected in series or parallel connections. For easy connection, three types of piezoelectric wafers with different electrode patterns are designed and fabricated. Moreover, based on the theory of piezo-elasticity, a simplified one-dimensional model is established to compare the electromechanical, transmitting and sensing performances of PiSSA actuators or sensors with series and parallel PZT wafers, and the model was validated by experiments.

The rest of the paper is organized as follows. Section 2 introduces the structure and fabrication procedure of PiSSAs with series and parallel wafers. Section 3 derives a simplified one-dimensional model for PiSSA actuators and sensors with series or parallel PZT wafers based on the piezo-elastic theory. Section 4 presents a comparative study of the electromechanical, transmitting and sensing performances of PiSSAs as well as the experimental validation. Section 5 concludes the paper.

## 2. Structure and Fabrication Procedure of PiSSAs

### 2.1. Structure of PiSSAs

**Figure 1:**
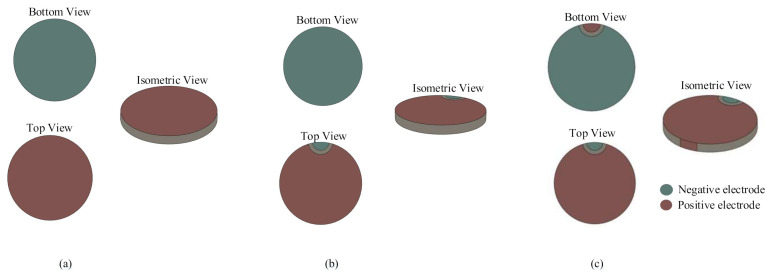
The structures of PZTs: (**a**) PZT-0; (**b**) PZT-1; and (**c**) PZT-2.

As shown in Figure 1, to be electrically connected easily, three types of PZT wafers (PZT-0, PZT-1 and PZT-2) with different electrode patterns are designed. As shown in Figure 1a, PZT-0 is the standard electrode pattern, and the positive and negative electrodes are placed on two surfaces individually. PZT-1, as shown in Figure 1b, is the wrap around electrode pattern, and the negative electrode is connected to the surface of the positive electrode. PZT-2, as shown in Figure 1c, is the double-side wrap around electrode pattern, and both the positive and negative electrodes are connected to the other side.

To protect PiSSAs and reduce the electromagnetic interference, a copper cylinder and cover are also designed.

### 2.2. Fabrication Procedure of PiSSAs

**Figure 2:**
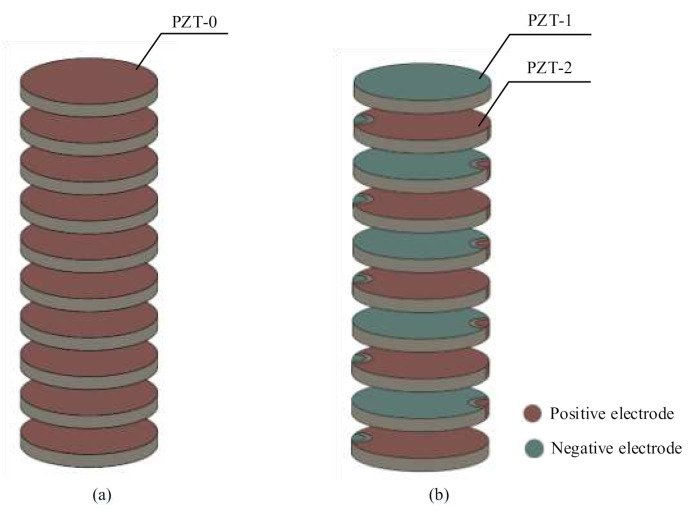
The structures of the PZT stack: (**a**) stack in series; and (**b**) stack in parallel.

Firstly, the individual PZT wafers are stacked together by conductive adhesive. As shown in Figure 2, the stack in series is connected by a number of PZT-0s, and the PZT wafers are directly electrically connected in series. Unlike the stack in series, the stack in parallel is comprised of PZT-1 and PZT-2, and the PZTs are electrically connected in parallel with the help of the designed electrode pattern. When the PZT wafers are stacked, the two adjacent PZT wafers are bonded by conductive adhesive and kept under a constant compression load of 150 N for 24 h.

**Figure 3:**
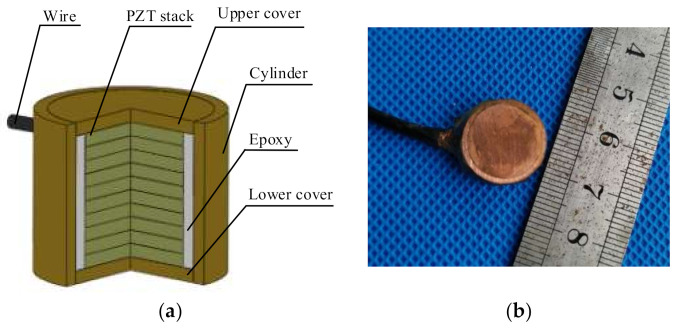
Structure of PiSSA: (**a**) structure of PiSSA; and (**b**) the assembled PiSSA.

Secondly, the wire is soldered to the cylindrical surface of the stack. Thirdly, as shown in Figure 3, the lower cover and the cylinder are firstly assembled using epoxy resin, and they are kept under the same compression load for another 24 h. In this step, the gap between the cylinder and the stack is filled with epoxy. At last, the upper cover is assembled and kept under the same load for 24 h.

## 3. Fundamentals of PiSSAs

### 3.1. Simplified Model of PiSSAs

**Figure 4:**
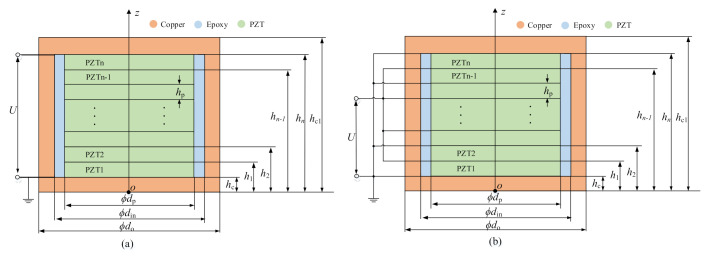
The simplified models of PiSSA actuators: (**a**) wafers in series connection; and (**b**) wafers in parallel connection.

**Figure 5:**
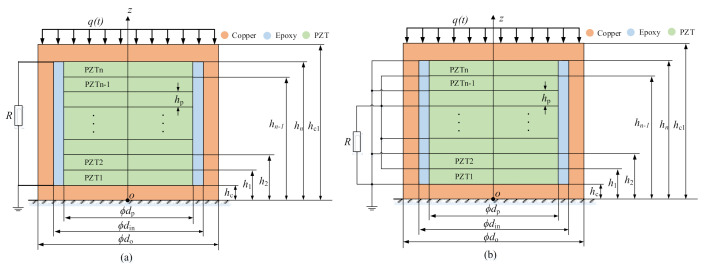
The simplified models of PiSSA sensors: (**a**) wafers in series; and (**b**) wafers in parallel.

Since PiSSAs are employed to transmit elastic stress waves in the axial direction, PiSSA actuators and sensors with series or parallel wafers are simplified to a one-dimensional axial model, as shown in Figure 4 and Figure 5, respectively. As shown in Figure 4, when an alternating voltage is inputted to PiSSA actuator, it excites an alternating strain on two surfaces of PiSSA, and PiSSA eventually excites ultrasounds in the concrete structures. As shown in Figure 5, when an alternating strain or stress is applied on the surface of PiSSA sensor, an alternating voltage is generated on the piezoelectric wafers.

In this model, *n* PZT wafers are connected in series or parallel. The thickness of the copper covers and PZTs are *h*_c_ and *h*_p_, respectively. The polarization direction of PZTs is along the *z*-axis, and the piezoelectric coefficient is positive if the polarization direction is along the positive *z*-axis; otherwise, it is negative. Note that the thickness and properties of the PZTs in PiSSAs are the same.

The fundamental equations for the *i*th composite layer of the PZT wafer, copper cylinder and epoxy are expressed as [65]

(1) $$\left\{\begin{array}{l} \sigma_{pi}=C_{33p}\varepsilon_{pi}-e_{33}E_{i}\\ D_{i}=e_{33}\varepsilon_{p_{i}}+\kappa_{33}^{\varepsilon }E_{i}\\ E_{i}=-\frac{\partial \phi_{i}}{\partial z}\\ \frac{\partial D_{i}}{\partial z}=0 \end{array}\right.\;\;i=1,\;2,\cdots ,\;n\;(\mathrm{PZT}\;\mathrm{wafer})$$

(2) $$\left\{\begin{array}{c} \sigma_{ci}=C_{33c}\varepsilon_{ci}\\ \sigma_{ei}=C_{33e}\varepsilon_{ei} \end{array}\right.\;\;\;i=1,\;2,\;\cdots ,\;n\;(\mathrm{copper}\;\mathrm{cylinder}\;\mathrm{and}\;\mathrm{epoxy})$$

where *σ*_c*i*_, *σ*_e*i*_ and *σ*_p*i*_, are the stresses of the copper, epoxy and the *i*th PZT wafer in the *z*-axis direction, respectively; *ε*_e*i*_, *ε*_c*i*_ and *ε*_p*i*_ are the strains of the copper, epoxy and the *i*th PZT wafer in the *z*-axis direction, respectively; *D_i_*, $\phi_{i}$ and *E_i_* are the electric displacement, electric potential and electric field of the *i*th PZT wafer, respectively; *C*_33c_, *C*_33p_ and *C*_33e_ are the Young’s moduli of the copper, the PZT wafer and the epoxy, respectively; and [65]

(3) $$\left\{\begin{array}{l} e_{33}=C_{33p}d_{33}\\ \kappa_{33}^{\varepsilon }=\kappa_{33}^{\sigma }-C_{33p}d_{33}^{2} \end{array}\right.$$

with $\kappa_{33}^{\sigma }$ and *d*_33_ the permittivity coefficient and piezoelectric coefficient, respectively.

In addition, the kinematic equation of the *i*th layer is expressed by

(4) $$\frac{\partial F_{pi}}{\partial z}=\left(\rho_{p}S_{p}+\rho_{e}S_{e}+\rho_{c}S_{c}\right)\frac{\partial^{2}u_{pi}}{\partial t^{2}}\;\;i=1,\;2,\;\cdots ,\;n$$

where *t* is the time; *u*_p*i*_ is the displacement in the *z*-axis direction of the *i*th layer; *F*_p*i*_ is the force applied on the *i*th layer in the *z*-axis direction; *ρ*_c_, *ρ*_e_ and *ρ*_p_ are the densities of the copper, the epoxy and the PZT materials, respectively; and $S_{p}=\pi d_{p}^{2}/4$, $S_{c}=\pi \left(d_{o}^{2}-d_{\mathrm{in}}^{2}\right)/4$ and $S_{e}=\pi \left(d_{p}^{2}-d_{\mathrm{in}}^{2}\right)/4$ are the cross-sectional area of the PZT wafer, the copper cylinder and the epoxy where din and do and are the inner and outer diameters of the copper cylinder respectively. *d*_p_ is the diameter of the PZT wafer.

Moreover, there are

(5) $$\left\{\begin{array}{l} F_{pi}=\sigma_{pi}S_{p}+\sigma_{ei}S_{e}+\sigma_{ci}S_{c}\\ \varepsilon_{pi}=\varepsilon_{ci}=\varepsilon_{ei}=\frac{\partial u_{pi}}{\partial z} \end{array}\right.\;\;i=1,\;2,\;\cdots ,\;n$$

Hence, combining Equations (1)–(5) yields

(6) $$\left\{\begin{array}{l} \chi_{0}\frac{\partial^{2}u_{pi}}{\partial z^{2}}=\chi_{1}\frac{\partial^{2}u_{pi}}{\partial t^{2}}\\ \frac{\partial E_{i}}{\partial z}=-\chi_{2}\frac{\partial^{2}u_{pi}}{\partial z^{2}} \end{array}\right.\;\;\;i=1,\;2,\;\cdots ,\;n$$

where the constants are defined by $\chi_{0}=S_{p}\left(C_{33p}+\chi_{3}\right)+S_{c}C_{33c}+S_{e}C_{33e}$, $\chi_{1}=S_{p}\rho_{p}+S_{c}\rho_{c}+S_{e}\rho_{e}$, $\chi_{2}=e_{33}/\kappa_{33}^{\varepsilon }$ and $\chi_{3}=e_{33}^{2}/\kappa_{33}^{\varepsilon }$.

Suppose that a harmonic voltage *U*(*t*) or a harmonic force *q*(*t*) is applied to PiSSAs and the harmonic load is given by

(7) $$\left\{\begin{array}{l} U(t)=U_{0}e^{j\omega t}\;\;\;\;(\mathrm{for}\;\mathrm{PiSSA}\;\mathrm{actutors})\\ q(t)=q_{0}e^{j\omega t}\;\;\;\;\;\;(\mathrm{for}\;\mathrm{PiSSA}\;\mathrm{sensors}) \end{array}\right.$$

where *U*_0_ and *q_0_* are the amplitude of the voltage and force, respectively; *j* is the unit imaginary number; and *ω* is the angular frequency.

For the harmonic and steady vibration, the displacement *u*_p*i*_ and electric potential $\phi_{i}$ at the *i*th layer are expressed by

(8) $$\left\{\begin{array}{l} u_{pi}=u_{pi}(z)e^{j\omega t}\\ \phi_{i}=\phi_{i}(z)e^{j\omega t}\\ F_{pi}=F_{pi}(z)e^{j\omega t} \end{array}\right.\;\;i=1,\;2,\;\cdots ,\;n$$

Hence, combining Equation (1) and Equations (6)–(8) gives the expression

(9) $$\left\{\begin{array}{l} u_{pi}(z)=A_{pi}\sin(k_{p}z)+B_{pi}\cos(k_{p}z)\\ \phi_{pi}(z)=\chi_{2}\left[A_{pi}\sin(k_{p}z)+B_{pi}\cos(k_{p}z)+C_{pi}z+D_{pi}\right]\\ F_{pi}(z)=\chi_{0}k_{p}\left[A_{pi}\cos(k_{p}z)-B_{pi}\sin(k_{p}z)\right]+\chi_{3}S_{p}C_{pi} \end{array}\right.\;\;\;\;\;i=1,\;2,\;\cdots ,\;n$$

where $k_{p}^{2}=\chi_{1}\omega^{2}/\chi_{0}$, *A*_p*i*_, *B*_p*i*_, *C*_p*i*_ and *D*_p*i*_, are constants which are determined by the mechanical and electric boundary conditions.

Similarly, the corresponding equations for the upper and lower copper covers are obtained

(10) $$\left\{\begin{array}{l} u_{ci}(z)=A_{ci}\sin(k_{c}z)+B_{ci}\cos(k_{c}z)\\ F_{ci}(z)=SC_{33c}k_{c}\left[A_{ci}\cos(k_{c}z)-B_{ci}\sin(k_{c}z)\right]\;\; \end{array}\right.\;\;\;\;\;i=1,2$$

where $\chi_{4}=\rho_{c}/c_{33c}$ and $k_{p}^{2}=\chi_{4}\omega^{2}$ are constants; $S=\pi d_{o}^{2}/4$ is the cross-sectional area of the copper cover; and *A*_c*i*_ and *B*_c*i*_ are the constants which are determined by the mechanical and electric boundary conditions of PiSSAs.

Therefore, there are totally 4 + 4 *n* variables in Equations (9) and (10), and the variables can be obtained by the mechanical and electric boundary conditions of PiSSAs.

### 3.2. Boundary Conditions of PiSSA Actuators

As shown in Figure 4, when a harmonic external voltage *U*(*t*) is input to PiSSA, it works as an actuator to transmit stress waves. Therefore, the mechanical boundary conditions of PiSSA actuators are given as

(11) $$\{\begin{array}{l} F_{c1}(z)|{}_{z=0}=0,\;F_{c2}(z)|{}_{z=h_{c1}}=0\\ F_{p1}(z)|{}_{z=h_{c}}=F_{c1}(z)|{}_{z=h_{c}}\\ F_{pn}(z)|{}_{z=h_{n}}=F_{c2}(z)|{}_{z=h_{n}}\\ F_{pi-1}(z)|{}_{z=h_{i-1}}=F_{pi}(z)|{}_{z=h_{i-1}}\\ u_{p1}(z)|{}_{z=h_{c}}=u_{c1}(z)|{}_{z=h_{c}}\\ u_{pn}(z)|{}_{z=h_{n}}=u_{c2}(z)|{}_{z=h_{n}}\\ u_{pi-1}(z)|{}_{z=h_{i-1}}=u_{pi}(z)|{}_{z=h_{i-1}} \end{array}\;\;\;i=2,\;3,\cdots ,\;n\;(\mathrm{for}\;\mathrm{PiSSA}\;\mathrm{actuators})$$

The electric current of the ith PZT in PiSSA actuators is given by

(12) $$I_{pi}(t)=\frac{dQ_{i}(t)}{dt}=\frac{d\int_{S_{p}}D_{i}(t)ds}{dt}=j\omega S_{p}e_{33}C_{pi}U_{0}e^{j\omega t}=I_{pi}(\omega )e^{j\omega t}$$

where $I_{pi}(\omega )=j\omega S_{p}e_{33}C_{pi}U_{0}$.

In addition, the electric boundary conditions for PiSSA actuators are expressed as

(13a) $$\left\{\begin{array}{l} \phi_{p1}(z)\left|{}_{z=h_{c1}}=0\right.\\ \phi_{pn}(z)\left|{}_{z=h_{n}}=U_{0}\right.\\ \phi_{pi-1}(z)\left|{}_{z=h_{i-1}}=\phi_{pi}(z)\left|{}_{z=h_{i-1}}\right.\right.\\ I_{p1}(\omega )=I_{p2}(\omega )=\cdots =I_{pn}(\omega ) \end{array}\right.i=2,\;3,\;\cdots ,\;n-1(\mathrm{for}\;\mathrm{PiSSAs}\;\mathrm{in}\;\mathrm{series})\;$$

or

(13b) $$\left\{\begin{array}{l} \phi_{p1}(z)\left|{}_{z=h_{c1}}=0\right.\\ \phi_{pn}(z)\left|{}_{z=h_{n}}=U_{0}\right.\\ \phi_{pi-1}(z)\left|{}_{z=h_{i-1}}=\phi_{pi}(z)\left|{}_{z=h_{i-1}}\right.\right.\\ I_{p1}(\omega )=I_{p2}(\omega )=\cdots =I_{pn}(\omega ) \end{array}\right.i=2,\;3,\;\cdots ,\;n-1(\mathrm{for}\;\mathrm{PiSSAs}\;\mathrm{in}\;\mathrm{parallel})\;$$

### 3.3. Boundary Conditions for PiSSA Sensors

As shown in Figure 5, when PiSSA works as sensors, an external uniform distributed force *q*(*t*) is applied on one side of PiSSA and the other side is fixed. Therefore, the mechanical boundary conditions are given as

(14) $$\left\{ \begin{array}{l} u_{c1}(z)\left|{}_{z=0}=0,\;F_{c2}(z)\left|{}_{z=h_{c1}}=q_{0}S\right.\right.\\ F_{p1}(z)\left|{}_{z=h_{c}}=F_{c1}(z)\left|{}_{z=h_{c}}\right.\right.\\ F_{pn}(z)\left|{}_{z=h_{n}}=F_{c2}(z)\left|{}_{z=h_{n}}\right.\right.\\ F_{pi-1}(z)\left|{}_{z=h_{i-1}}=F_{pi}(z)\left|{}_{z=h_{i-1}}\right.\right.\\ u_{p1}(z)\left|{}_{z=h_{c}}=u_{c1}(z)\left|{}_{z=h_{c}}\right.\right.\\ u_{pn}(z)\left|{}_{z=h_{n}}=u_{c2}(z)\left|{}_{z=h_{n}}\right.\right.\\ u_{pi-1}(z)\left|{}_{z=h_{i-1}}=u_{pi}(z)\left|{}_{z=h_{i-1}}\right.\right. \end{array}i=2,\;3,\cdots ,\;n\;(\mathrm{mechanical}\;\mathrm{boundary}\;\mathrm{conditions})\right.$$

Similarly, the electric boundary conditions for PiSSA sensors are expressed as

(15a) $$\left\{\begin{array}{l} \phi_{p1}(z)\left|{}_{z=h_{c1}}=0\right.\\ \phi_{pn}(z)\left|{}_{z=h_{n}}=I_{pi}(\omega )R\right.\\ \phi_{pi-1}(z)\left|{}_{z=h_{i-1}}=\phi_{pi}(z)\left|{}_{z=h_{i-1}}\right.\right.\\ I_{p1}(t)=I_{p2}(t)=\cdots =I_{pn}(t) \end{array}\right.i=2,\;3,\;\cdots ,\;n-1\;(\mathrm{for}\;\mathrm{PiSSAs}\;\mathrm{in}\;\mathrm{series})\;$$

or

(15b) $$\left\{\begin{array}{l} \phi_{p1}(z)\left|{}_{z=h_{c}}=\phi_{pi}(z)\left|{}_{z=h_{pi}}=\phi_{pi+1}(z)\left|{}_{z=h_{pi}}=0\;\;\;\;\;i=2,\;4,\;6\cdots \right.\right.\right.\\ \phi_{pn}(z)\left|{}_{z=h_{n}}=\phi_{pi}(z)\left|{}_{z=h_{i}}=\phi_{pi+1}(z)\left|{}_{z=h_{i}}=R\sum_{i}^{n}I_{pi}(\omega )\right.\;\;\;i=3,\;5,\;7\cdots \right.\right. \end{array}\right.(\mathrm{for}\;\mathrm{PiSSAs}\;\mathrm{in}\;\mathrm{parallel})\;$$

where *R* is the resistor of the closed electric circuit.

### 3.4. Analytical Solutions to PiSSAs

Combining Equations (9)–(15), 4 + 4 *n* equations are totally obtained for a given frequency *ω,* and the 4 + 4 *n* variables in Equations (9) and (10) can be solved by these 4 + 4 *n* equations by numerical computation with the help of a computer.

As a result, the impedance of PiSSA actuators for a given angular frequency *ω* is given by

(16) $$Z(\omega )=\left\{\begin{array}{l} \frac{U(t)}{I_{pi}(t)}=\frac{U_{0}}{I_{pi}(\omega )}\;\;\;\;\;\;\;\;\;\;(\mathrm{for}\;\mathrm{PiSSAs}\;\mathrm{in}\;\mathrm{series})\\ \frac{U(t)}{\sum_{i}^{n}I_{pi}(t)}=\frac{U_{0}}{\sum_{i}^{n}I_{pi}(\omega )}\;\;\;(\mathrm{for}\;\mathrm{PiSSAs}\;\mathrm{in}\;\mathrm{parallel}) \end{array}\right.$$

Hence, the electromechanical coupling factor of PiSSAs can be obtained by [81]

(17) $$k^{2}=\frac{f_{a}^{2}-f_{r}^{2}}{f_{a}^{2}}$$

where *f*_r_ and *f*_a_ are the resonance and anti-resonance frequencies which can be obtained by Equation (16).

The voltage output of PiSSA sensors is given by

(18) $$U(\omega )=\left\{\begin{array}{l} I_{pi}(\omega )R\;\;\;\;\;\;\;\;\;\;(\mathrm{for}\;\mathrm{PiSSAs}\;\mathrm{in}\;\mathrm{series})\\ R\sum_{i}^{n}I_{pi}(\omega )\;\;\;\;\;\;(\mathrm{for}\;\mathrm{PiSSAs}\;\mathrm{in}\;\mathrm{parallel}) \end{array}\right.$$

In addition, the average power of PiSSAs is expressed as

(19) $$P(\omega )=\left\{\begin{array}{l} U(\omega )I_{pi}(\omega )\;\;\;\;\;\;\;\;\;\;(\mathrm{for}\;\mathrm{PiSSAs}\;\mathrm{in}\;\mathrm{series})\\ U(\omega )\sum_{i}^{n}I_{pi}(\omega )\;\;\;\;\;\;(\mathrm{for}\;\mathrm{PiSSAs}\;\mathrm{in}\;\mathrm{parallel}) \end{array}\right.$$

## 4. Performance Comparison of PiSSAs

### 4.1. Parameters of PiSSAs

After the simplified model is established, the electromechanical, transmitting and sensing performance of PiSSAs whose wafers are electrically connected in series and parallel are compared in this section. The structural dimensions and material parameters are listed in Table 1 and Table 2, respectively.

### 4.2. Electromechanical Performance

**Figure 6:**
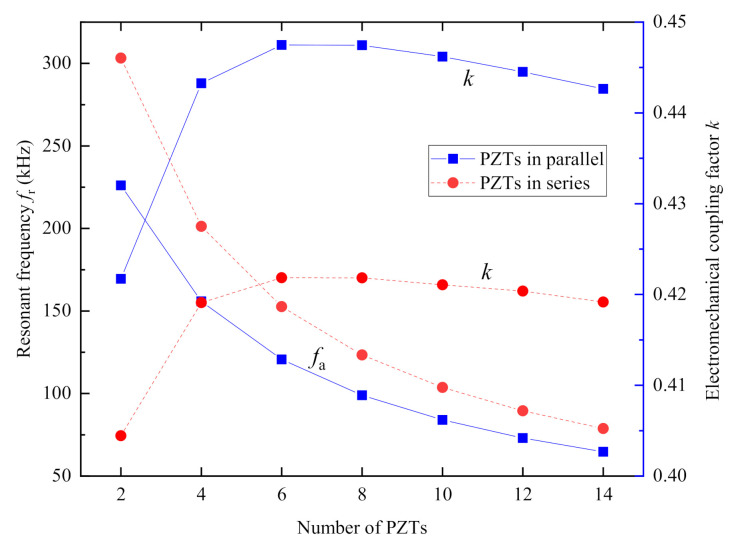
The first resonance frequency *f*_r_ and electromechanical factor *k* of PiSSAs versus the number of the connected PZT wafers.

Figure 6 plots the curves of the first resonance frequency *f*_r_ and the electromechanical coupling factor *k* versus the number of the PZT wafers *n*. Figure 6 indicates that the first resonance frequency *f*_r_ of both PiSSAs with series and parallel connected wafers decreases as the number of the PZT wafers *n* increases while the first electromechanical coupling factor increases firstly and then decreases gradually as the number increases. Figure 6 also shows that the first resonance frequency *f*_r_ of PiSSAs with series wafers is about 1.21–1.34 times that in the case of parallel wafers, and the electromechanical coupling factor *k* of PiSSAs with series wafer is about 0.94–0.96 times that in parallel. Moreover, the first electromechanical coupling factor reaches the peak when the number of the PZT wafers is 6 for the given structural parameters, which indicates that there is an optimal value for the number of the PZT wafers for a given structure. Since PiSSA, as shown in Figure 3a, mainly consists of a copper cylinder, an upper cover, a lower cover, a piezoelectric stack and epoxy, the height of the copper cylinder and epoxy increases as the number of piezoelectric wafers increases, and they dissipate more energy. Moreover, the piezoelectric wafers transfer more mechanical energy from electrical energy as the number increases. When the change of the energy dissipated by the structure is less than the change of the energy transferred by the stack, the electromechanical coupling factor increases, and, on the other side, the factor decreases. As a result, the first electromechanical coupling factor increases firstly and then decreases gradually as the number increases, and there is an optimal number where the first electromechanical coupling factor reaches the maximum value.

**Figure 7:**
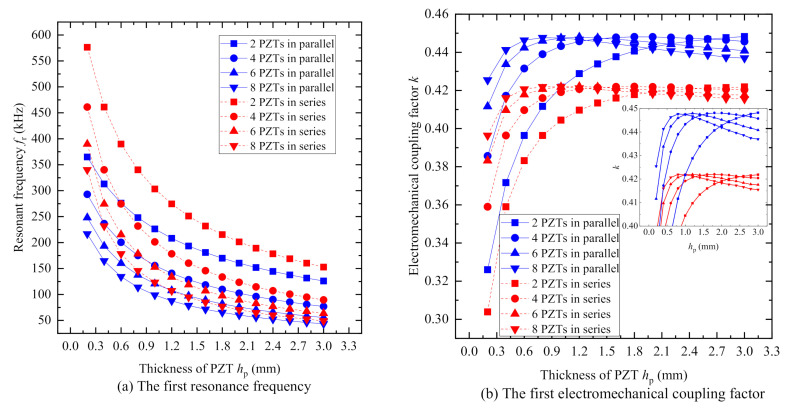
The first resonance frequency *f*_r_ and electromechanical factor *k* of PiSSAs versus the thickness of PZT wafer: (**a**) the first resonance frequency; and (**b**) the first electromechanical coupling factor.

Figure 7 shows the curves of the first resonance frequency *f*_r_ and electromechanical coupling factor of PiSSAs versus the thickness of the PZT wafer *h*_p_, respectively. Figure 7a reveals that the first resonance frequencies of PiSSAs with series or parallel wafers decrease as the thickness of the PZT wafer *h*_p_ increases. Figure 7b illustrates that the first electromechanical coupling factor firstly increases and then decreases as the thickness *h*_p_ increases, and the first electromechanical coupling factor in parallel is slightly larger than that with series wafers, which indicates that there is an optimal value for the thickness hp of the PZT wafers for a given structure.

**Figure 8:**
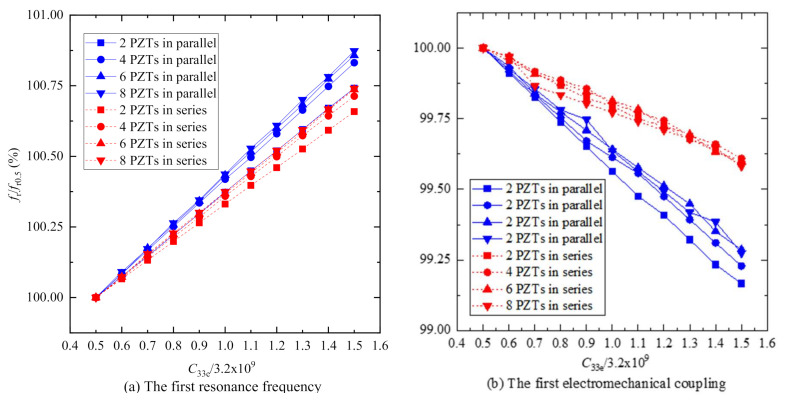
The first resonance frequency *f*_r_ and electromechanical factor *k* of PiSSAs versus the Young’s modulus of the epoxy *C*33e (*f*_r0.5_ and *k*_0.5_ are the first resonance frequency and electromechanical factor when the Young’s modulus is 0.5 times of 3.2 GPa): (**a**) the first resonance frequency; and (**b**) the first electromechanical coupling factor.

Figure 8 plots the curves of the first resonance frequency *f*_r_ and electromechanical coupling factor *k* versus the Young’s modulus of the epoxy *C*_33e_. Figure 8a reveals that the first resonance frequency *f*_r_ of PiSSA with series wafers just increases 0.74% as the Young’s modulus *C*_33e_ increases from 0.5 to 1.5 times 3.2 GPa while the frequency of PiSSA with parallel wafer increases 0.87%. Similarly, Figure 8b illustrates that the first electromechanical coupling factor *k* of PiSSA with series wafers decreases 0.42% as the Young’s modulus *C*_33e_ increases from 0.5 to 1.5 times 3.2 GPa while the factor of PiSSA with parallel wafers decreases 0.83%. Therefore, the curves in Figure 8 indicates that the Young’s modulus of the epoxy *C*_33e_ has little effect on the first resonance frequency *f*_r_ and electromechanical coupling factor *k*, which can be helpful for the design of PiSSAs.

### 4.3. Transmitting Performance

**Figure 9:**
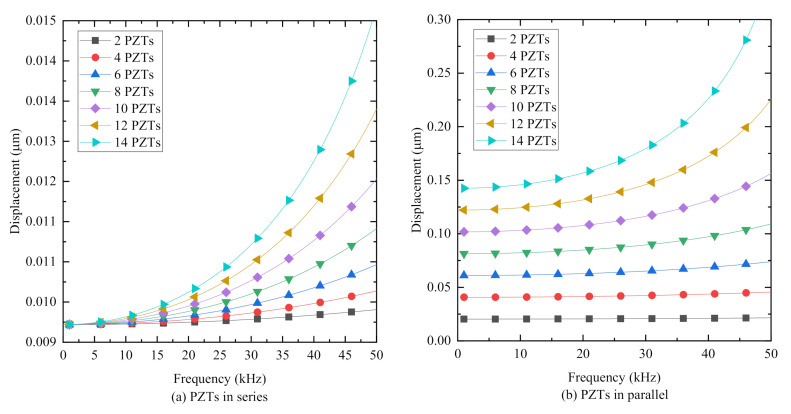
The displace at *z* = *h*_c1_ versus the number of the PZT wafers *n:* (**a**) PZTs in series connection; and (**b**) PZTs in parallel connection.

Figure 9 plots the curves of the displacement of PiSSAs with series or parallel wafers under an excitation voltage with an amplitude of 100 V versus the frequency of the excitation voltage. Figure 9 illustrates that the displacement of PiSSAs with series or parallel wafers increases as the number of the connected PZTs increases, and the displacement also increases as the frequency increases. Compared with Figure 9b, Figure 9a shows that the displacement of PiSSAs with parallel wafer is about 2.18–22.49 times that with series wafers, which indicates that PiSSAs in parallel are much more suitable for the use as actuators to excite stress waves. Moreover, there are only one or two wafers in the conventional SA, and, therefore, PiSSA actuators with more piezoelectric wafers generate a larger output and display a better transmitting performance than the conventional one.

### 4.4. Sensing Performance

Since the output voltage is the most important property of PiSSA sensors, the voltage is compared in this section and the voltage is obtained by Equation (18).

**Figure 10:**
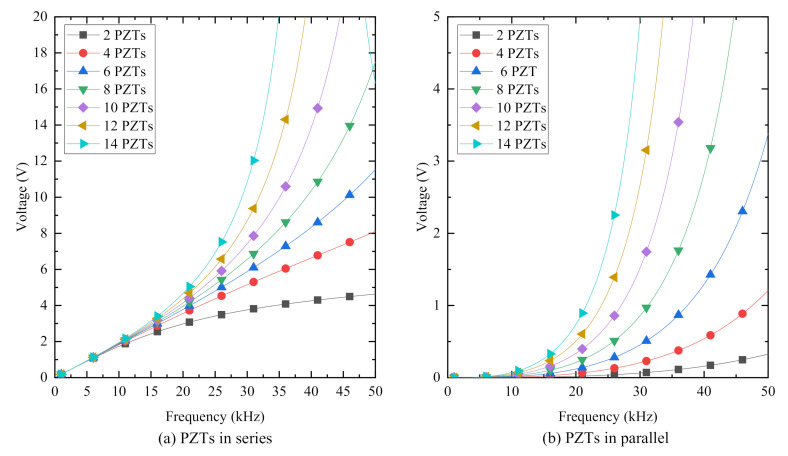
The output voltage of PiSSAs with a resistor R = 10 kΩ versus the frequency of the excitation load: (**a**) PZTs in series connection; and (**b**) PZTs in parallel connection.

Figure 10 plots the curves of the output voltage of PiSSAs in series and parallel connection with an external resistor 10 kΩ under a uniform distributed load with an amplitude of 0.1 MPa versus the frequency of the excitation load. Figure 10 reveals that the voltage of PiSSAs with wafers in series or parallel connection increases as the number of the connected PZTs increases, and the voltage also increases as the frequency increases. Compared with Figure 10b, Figure 10a shows that the output voltage of PiSSAs with series wafers is much larger than that of PiSSAs with parallel wafers, which indicates that PiSSAs with series wafer are much more suitable for the use as sensors to detect stress waves. Moreover, there are only one or two wafers in the conventional SA, and the two wafers are usually connected in parallel. Therefore, PiSSA sensors with more piezoelectric wafers output a larger voltage and show a better sensing performance than the conventional one.

It should be noted that the output voltage of PiSSAs may vary as the external matching resistor changes, and the external matching resistor should be chosen carefully [82].

### 4.5. Experimental Validation

**Figure 11:**
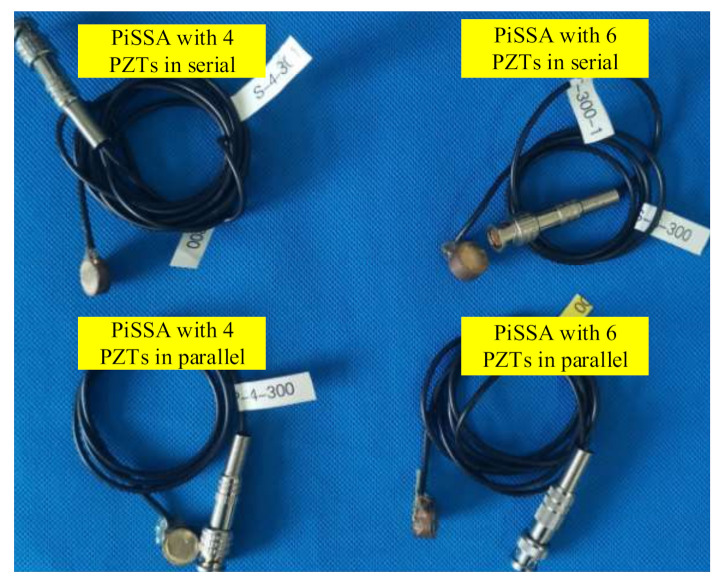
The four fabricated PiSSAs.

**Figure 12:**
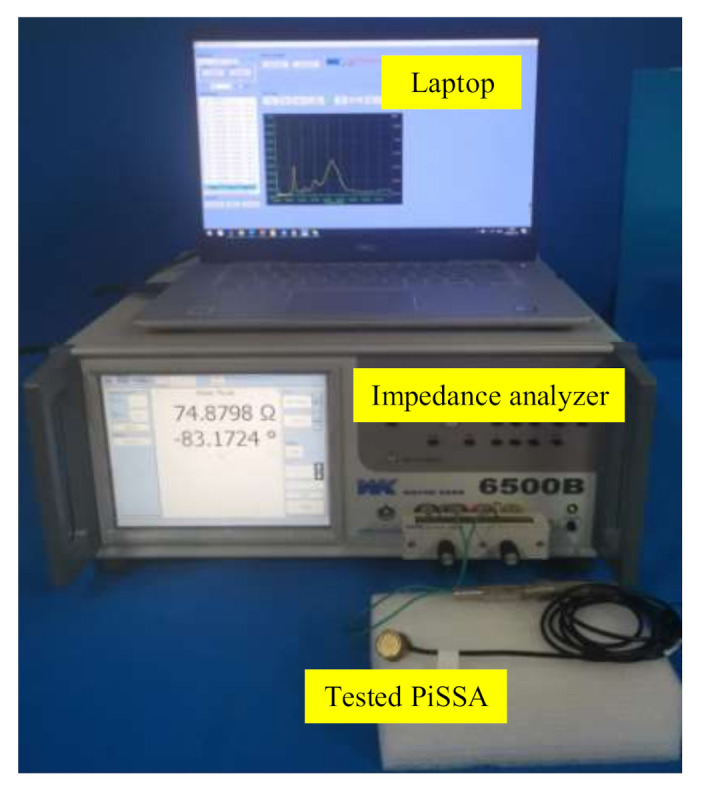
Experimental setup.

To validate the simplified model of PiSSAs, four PiSSAs including two PiSSAs with series wafers and two PiSSAs with parallel wafers (Figure 11) were fabricated and their impedance signatures were measured by an impedance analyzer. To decrease the adverse effect of the fabrication process of PiSSAs with too many piezoelectric wafers, the number of the PZT wafers were 4 and 6, respectively. As shown in Figure 12, the measured experimental setup included an impedance analyzer (Wayne Kerr 6500B), PiSSAs and a laptop.

**Figure 13:**
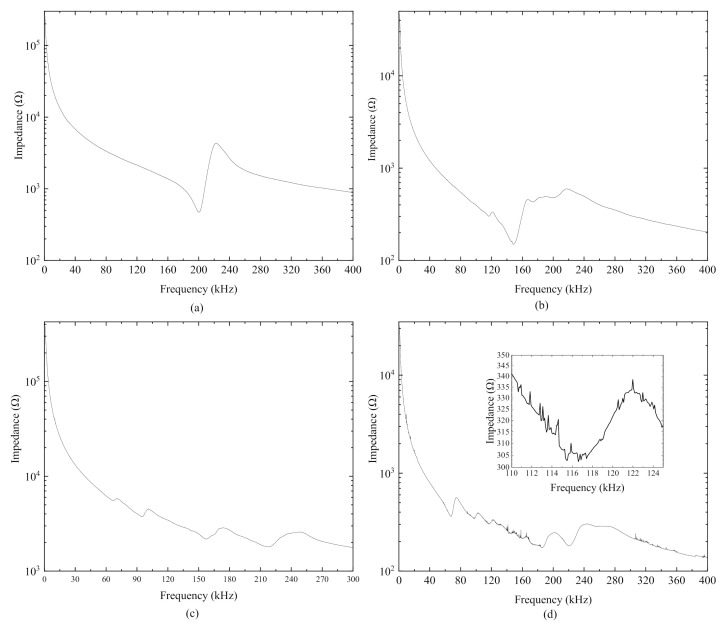
The measured impedances of PiSSAs with different number of PZT wafers in series or parallel connection: (**a**) four series PZT wafers; (**b**) four parallel PZT wafers; (**c**) six series PZT wafers; and (**d**) six parallel PZT wafers.

Figure 13 plots the measured impedance signatures of PiSSAs, and, due to the effect of the conductive adhesive and soldering, some noises are observed in the figures, especially in Figure 13d. Table 3 compares the measured and the theoretical first resonance and anti-resonance frequencies of the four fabricated PiSSAs. Table 3 reveals that the theoretical values of the first resonance and anti-frequencies are in good agreement with the experimental ones, and the relative error is about 0.40–10.57%. Therefore, the experimental results validate that the simplified model for PiSSAs can be used to study the performance of PiSSAs.

It should be noted that the conductive adhesive between the adjacent two piezoelectric wafers is not considered in the simplified model, which may bring about additional noise and error between the experimental and theoretical data as the number of the piezoelectric wafers increases. Moreover, the features of PiSSA sensors, such as the repeatability, noise/signal ration, hysteresis behavior, etc., are also important and should be tested.

### 4.6. Discussion

Based on the simplified model of PiSSAs, the electromechanical, transmitting and sensing performance of PiSSAs with series and parallel wafers are compared. The theoretical results indicate that the first resonance frequency and the first electromechanical coupling factor are influenced by the number, electrical connecting type and thickness of the PZT wafers. The displacement and voltage output of PiSSAs are influenced greatly by the connecting type and number of the PZT wafers. Therefore, the connecting type, number and thickness of the PZT wafers should be carefully selected to increase the efficiency and output of PiSSA actuators or sensors when PiSSAs are designed. This study also contributes to provide a method to optimize the structural parameters of PiSSA sensors and actuators in our future work.

Moreover, four PiSSAs including two PiSSAs with series wafers and two PiSSAs with parallel wafers were fabricated, and the measured electromechanical data of the four PiSSAs are in good consistency with the theoretical one.

## 5. Conclusions

A novel piezoceramic stack-based smart aggregate (PiSSA) is proposed to improve the efficiency and output performance over the traditional SAs with only one piezoceramic patch. PiSSA is especially applicable to the situations where the signal easily attenuates. In PiSSA, the piezoelectric wafers are electrically connected in series or parallel. For easy series or parallel connection, three types of PZT wafers with different electrode patterns are designed. Based on the theory of piezo-elasticity, a simplified one-dimensional model is established to investigate the characteristics of the proposed PiSSAs. By using this simplified model, the electromechanical, transmitting and sensing characteristics of PiSSAs with series and parallel wafers are theoretically investigated, and the simplified model was also validated by experiments. The following conclusions are drawn:

(1) The first resonance frequency of PiSSAs where the wafers are in series or parallel connection decreases as the number and thickness of the PZT wafers increases, and the first electromechanical coupling factor increases firstly and then decreases gradually as the number and thickness increases, which reveals that there is an optimal number and thickness for the electromechanical coupling factor.

(2) The first resonance frequency and the first electromechanical coupling factor of PiSSA where the wafers are in series or parallel connection changes no more than 0.87%, as the Young’s modulus of the epoxy increases from 0.5 to 1.5 times 3.2 GPa, which can be helpful for the fabrication of PiSSAs.

(3) The displacement output of PiSSAs with parallel wafers is about 2.18–22.49 times that of the case of series wafers within 1–50 kHz, while the voltage output of PiSSAs with parallel wafers is much less than that of PiSSAs with series wafers, which indicates that PiSSAs with parallel wafers are much more suitable for working as actuators to excite stress waves and PiSSAs in series are suitable for working as a sensor to detect the waves.

This study contributes to providing a method to investigate the performance of PiSSAs. The future work will involve the structural parameters and external matching resistor optimization, and the transmitting and sensing performance test of PiSSAs. The future work will also involve the theoretical and experimental study of PiSSA sensor features, such as repeatability, hysteresis behavior, etc.

## Figures and Tables

**Table 1:** The structural dimension and material type of PiSSA.

	Dimensions (mm)	Material Type
PZT wafer	*d*_p_ = 10, *h*_p_ = 1	PZT−5
Upper and Lower Covers	*h*_c_ = 1	Copper
Cylinder	*d*_o_ = 14, *d*_in_ = 12	Copper
Epoxy	/	Pattex PKM12C−1

**Table 2:** The material parameters of PiSSA.

	PZT−5	Copper	Epoxy
Density *ρ* (kg/m^3^)	7450	8800	1650
Young’s modulus *C*_33_ (GPa)	90	90	3.2
*d*_33_ (pC/N)	420	/	/
$\kappa_{33}^{\sigma }/\varepsilon_{0}$	2200	/	/

$\varepsilon_{0}=8.85\times 10^{-12}\;F/m$, permittivity of free space.

**Table 3:** Comparison of the first resonance and anti-resonance frequencies of PiSSAs.

	Resonance Frequency (kHz)			Anti-resonance Frequency (kHz)		
	Measured	Theoretical	Relative Error	Measured	Theoretical	Relative Error
Four PZTs in series	202.8	201.29	0.74	222.6	221.7	0.40
Six PZTs in series	156.3	152.61	2.36	173.8	168.32	3.15
Four PZTs in parallel	148.5	155.81	4.92	165.3	173.82	5.15
Six PZTs in parallel	116.6	120.64	3.46	122.0	134.9	10.57
